# The Bright Side of Abstraction: Abstractness Promoted More Empathic Concern, a More Positive Emotional Climate, and More Humanity-Esteem After the Paris Terrorist Attacks in 2015

**DOI:** 10.3389/fpsyg.2020.545662

**Published:** 2020-11-26

**Authors:** Itziar Fernández, Amparo Caballero, Verónica Sevillano, Dolores Muñoz, Luis Oceja, Pilar Carrera

**Affiliations:** ^1^Department of Social and Organizational Psychology, Faculty of Psychology, National Distance Education University, Madrid, Spain; ^2^Department of Social Psychology and Methodology, Faculty of Psychology, Autonomous University of Madrid, Madrid, Spain

**Keywords:** empathic concern, emotional climate, terrorist attack, abstraction, humanity-esteem

## Abstract

**Antecedents:**

Previous research on citizens’ reactions after terrorist events has shown that positive reactions can also emerge alongside pain and horror. Positive emotions have been widely associated with an abstract style of thinking. In the context of the Paris terrorist attacks in 2015, we explored Spanish citizens’ positive reactions – empathic concern, positive emotional climate, and esteem for humanity – and examined the relationships of these responses with an abstract (vs. concrete) style of thinking.

**Method:**

A longitudinal study was designed involving an online questionnaire that was administered 10 days, 3 weeks, and 2 months after the attacks (*N* = 253).

**Results:**

Empathic concern and personal distress toward Parisians decreased from the weeks following the attacks to 2 months later, with empathic concern always being more intense than personal distress. Emotional climate was perceived as more hostile than positive, although positive feelings persisted. People reported moderately positive esteem for humanity. Individuals with a more abstract style of thinking reported greater empathic concern, a more positive emotional climate, and more esteem for humanity.

**Conclusions:**

Our results support and extend previous research showing that abstraction enhances people’s resilience, even under traumatic circumstances such as those surrounding a terrorist attack.

## Introduction

Since September 2001 event in New York and Washington DC, a large body of research has addressed how people deal with collective violence, such as terrorist attacks. After the attacks in the United States, Europe suffered the terrible pain caused by terrorists on several occasions: Madrid (2004), London (2005, 2017), Oslo and Utøya (2011), Paris (2015), Berlin, Brussels, Nice (2016), Stockholm, Manchester, Barcelona (2017), and Strasbourg (2018). All of these dramatic events have created countless direct and indirect victims.

After the massacre at the Charlie Hebdo journal office in Paris in January 2015, terror hit Paris again with six simultaneous attacks during November 2015, leaving 130 dead and more than 300 injured. News about these attacks were followed by thousands of people worldwide. The media and social networks allowed people to share information about the terrible damage suffered by the victims; communication that fostered a wave of solidarity and support around the world toward Parisians. Indeed, such social communication promotes emotional reactions in people who are not directly affected by the attacks (i.e., *indirect* or *vicarious victims*). These vicarious victims may also suffer negative emotional consequences (e.g., Pelletier and Drozda-Senkowska, 2016; Luhmann and Bleidorn, 2018) and even major depressive disorders (e.g., Salguero et al., 2011).

Solidarity with France and the French was displayed through hundreds of individual responses and collective demonstrations around the world. Emotional, cognitive, and behavioral reactions after terrorist attacks have been widely studied (e.g., Mehl and Pennebaker, 2003; Conejero and Etxebarría, 2007; Páez et al., 2007; Fernández et al., 2009; McArdle et al., 2012), also in the case of Paris (e.g., Pelletier and Drozda-Senkowska, 2016; Luhmann and Bleidorn, 2018; Garcia and Rimé, 2019). Such previous research shows that the emotions caused by a terrorist event significantly affect not only individuals’ psychological reactions but also their social lives in the form of social sharing (Fernández et al., 2009; Garcia and Rimé, 2019) and collective reactions (Páez et al., 2007; McArdle et al., 2012; Pelletier and Drozda-Senkowska, 2016). Negative emotions (fear, anger, sadness, anxiety, and sorrow) are most frequent after terrorist acts (Conejero and Etxebarría, 2007; Bux and Coyne, 2009; Giner-Sorolla and Maitner, 2013). However, longitudinal research has shown that this negative mood declines over time (e.g., Fernández-Dols et al., 2007; Fernández et al., 2009; Pelletier and Drozda-Senkowska, 2016; Luhmann and Bleidorn, 2018).

The consequences of terrorism are not only negative. Citizens are capable of overcoming the drama by developing prosocial behaviors (Pérez-Sales et al., 2005) and positive emotions, such as energy, optimism, strength, pride, or joy (Smith et al., 2001; Fredrickson et al., 2003; Fernández-Dols et al., 2007; Fernández et al., 2009; Vázquez and Hervás, 2010; Pelletier and Drozda-Senkowska, 2016). For example, the general population did not show changes in their benevolent views of the world after March 2004 bombings in Madrid (Ubillos et al., 2005). These positive reactions open the door to encouraging constructive responses after terror events. Positive feelings facilitate creative solutions, providing individuals with an advantage to overcome obstacles and difficulties (Fredrickson, 2013). This cognitive broadening implies taking a more abstract perspective that improves creativity, self-control, well-being, and cooperative problem solving (see for a review, Burgoon et al., 2013).

Victims of traumatic events usually see the world as uncontrollable, threatening, and malevolent (Janoff-Bulman, 1992). However, being able to construe a traumatic situation in a positive way while controlling immediate negative reactions allows people to successfully face the present and focus on the future. Among the several psychological mechanisms that may promote these healthy reactions, we focus on the construal level. Construal level theory (CLT, Liberman and Trope, 1998; Trope and Liberman, 2003) points out that people can subjectively represent or construe events differently depending on their style of thinking or construal level. Mental representations can vary from a concrete style focused on the near situation to an abstract style focused on the distant future (Trope and Liberman, 2003). The personal construal level is a dispositional trait that can be modified by situational cues, such as difficulty of the action (see Vallacher and Wegner, 1989) or psychological distance (see Trope and Liberman, 2003). Differences in how individuals represent a situation have important consequences on their judgments and decisions (Fujita, 2008), and this reasoning can be applied to terrorist acts and their consequences. In this sense, when an individual presents an abstract construal level, she/he mentally represents the events by focusing on future goals; a style of thinking that motivates them to overcome obstacles and difficulties (Vallacher and Wegner, 1989; Liberman and Trope, 1998; Carrera et al., 2017, 2019).

Therefore, if we look at the immediate damage caused by terrorism (concrete style) instead of thinking about the future recovery (abstract style), we are more likely to fall into a negative mood. In contrast, focusing on overcoming difficulties in the future (abstract style) promotes a positive approach that is more beneficial to individuals and society. Indeed, an abstract construal level focused on the distant future has shown to buffer negative events in one’s daily life, improving self-esteem and well-being (Updegraff et al., 2010). Regarding terrorist attacks, research has shown that individuals who held higher power positions (e.g., government officials) construed the aftermath of September 2001, more abstractly and positively (Magee et al., 2010). These results support that an abstract construal level leads people to focus less on negative details and reinterpret facts as an opportunity for personal growth.

In the present work, we state that, when faced with such a dramatic event, such as the outburst of a terrorist attack, the disposition to keep an abstract construal level has positive consequences at both the interpersonal and collective levels.

### Present Study

Research previously described the influence of terrorist acts on intrapersonal (e.g., mood) and collective feelings (e.g., emotional climate). We extend the study to the interpersonal level by focusing on the vicarious emotions reported when perceiving victims. As main vicarious emotions, Batson (2011) pointed out empathic concern (e.g., feeling moved, compassionate, and warm) and personal distress (e.g., feeling alarmed, worried, and distressed). Empathic concern is an other-oriented emotion in the sense that it involves feeling concerned for the other, whereas personal distress involves feeling distressed by the state of the other (self-oriented emotion). The distinction between *for* and *by* is important in explaining the social motives associated with each vicarious emotion: motivation evoked by empathic concern is altruistic because the ultimate goal is to reduce the other’s need, whereas the motivation evoked by personal distress is egoistic because the goal is to reduce one’s own aversive arousal (Batson, 2011). These two vicarious emotions are qualitatively distinct, presenting empathic concern a more positive valence and lower arousal than personal distress (López-Pérez et al., 2014). After the terrorist attacks in 2015, Parisians were prototypical targets of this mixed vicarious emotional experience formed by empathic concern and personal distress. Analyzing the emotion that prevails after a terrorist attack is interesting because empathic concern can be generalized in multiple-victim situations, promoting the helping of each people in need (see Oceja et al., 2017).

Whereas we measure empathic concern and personal distress at the interpersonal level, we evaluate emotional climate (de Rivera and Páez, 2007) and esteem for humanity (Luke and Maio, 2009) at the collective level. The emotional climate refers to the predominant collective emotions generated through the social interaction of group members at a particular time (de Rivera and Páez, 2007). Perceiving the emotional climate as positive after a terrorist attack predicts social support and posttraumatic growth (Basabe et al., 2004; Páez et al., 2007). Esteem for humanity refers to the extent to which individuals perceive humans in general as favorable, desirable, competent, and trustworthy (Luke and Maio, 2009). Humanity-esteem can be altered after experiencing a stimulus that threatens important social values (Luke and Maio, 2009). Terrorist attacks can deeply affect our core assumptions about the benevolence of people and the world (Janoff-Bulman, 1992; Vázquez et al., 2008), and offer a traumatic but challenging opportunity to study this change.

As a novelty, we examine the role played by the abstract construal level on vicarious emotions, collective feelings, and attitudes. The present study followed a longitudinal perspective to explore the temporal changes in Spaniards’ emotional reactions toward Parisians after the terrorist attacks of November 2015.

First, we tested whether the intensity of empathic concern and personal distress toward Parisians declined over time as previous research has found when measuring general mood and well-being after acts of terror. Second, we examined whether the link previously observed between abstraction and intrapersonal positive emotions extends to vicarious and collective reactions after the terrorist attacks. We expected that individuals higher in abstraction would report greater empathic concern and a more positive emotional climate and attitude toward humanity. Because previous research on abstraction has not addressed its direct influence on negative reactions, we did not make specific predictions relating abstraction with personal distress and negative emotional climate.

## Materials and Methods

### Participants

The sample comprised 253 psychology students at the National Open University in Spain, who participated in exchange for course credit (196 females; *M*_age_ = 35.98; SD = 9.30). The study was conducted as part of a survey that included other measures unrelated to the hypotheses raised in the present research. At the beginning of the survey, the participants were randomly assigned to follow one of two different sets of instructions. Some participants (*N* = 178) wrote about terrorist attacks (main condition) in the first and second sessions (focusing on Paris in the first session and on France and Europe in the second session). The other participants (*N* = 75) wrote about a personal social event (control condition) in both the first and the second session. In the last session, the participants did not write narratives. Participants were randomized to each type of writing, but the number of participants differed because the control condition was included 3 days later as a result of technical problems. The writing manipulation did not influence the measures (construal level, empathic concern, personal distress, emotional climate and esteem for humanity) reported in this paper (*F*s ≤ 1.79, *p*s > 0.05). For this reason, the data were combined.

### Procedure

Following the longitudinal approach introduced by Pennebaker and Harber (1993), the participants were asked to complete the scales in three different sessions: Time 1 on approximately November 25, 2015; Time 2 on approximately December 2, 2015; and Time 3 on approximately January 13, 2016. We spent 12 days organizing the data collection after the attacks. The participants were asked to focus on Parisians after the terrorist attacks in November 2015 (when citizens were injured and threatened by terrorism) as they answered the questions on the empathic concern and personal distress scales; these vicarious emotions were measured in all sessions. The emotional climate and esteem for humanity scales were evaluated only in the last session. In the last session, participants also completed a dispositional measure of abstraction and reported their age and sex.

We note that the participants completed the emotional climate scale and esteem for humanity scale after their reporting empathic concern and personal distress toward Parisians after the terrorist attacks. This order was designed to focus participants on the traumatic event that occurred in Paris when reporting their collective feelings and attitudes.

### Measures

#### Empathic Concern and Personal Distress

Two indexes adapted and validated to the Spanish context (Oceja and Jiménez, 2007) from the Empathic Response Questionnaire (ERQ, Batson et al., 1983) were used to measure situational vicarious emotions focused on Parisians after the terrorist attacks. The empathic concern index included the following terms: *warm, tender, moved, compassionate, softhearted*, and *I feel sorry for them* (Cronbach’s alphas: α_Time 1_ = 0.86; α_Time 2_ = 0.90; α_Time 3_ = 0.92). The personal distress index included the following terms: *troubled, agitated, distressed, upset, worried*, and *disturbed* (Cronbach’s alphas: α_Time 1_ = 0.81; α_Time 2_ = 0.89; α_Time 3_ = 0.88). The participants were asked, “*Right now, knowing about the Paris terrorist attacks, to what extent are your feelings toward Parisians (emotional term)?*” They were asked to rate each item on a Likert-type scale ranging from 1 (*not at all*) to 7 (*very much*). Higher scores indicated higher emotional intensity.

#### Emotional Climate

The participants’ perceptions of positive and negative collective emotions were measured with the emotional climate scale (Páez et al., 1997). The participants reported their agreement with eleven items (e.g., “*The social environment or climate is one of hope*”) on Likert-type scale ranging from 1 (*not at all*) to 5 (*very much*). The positive emotions included *goodness, peace, joy, hope, solidarity, bonanza, and confidence* (α_Time 3_ = 0.80), and the negative emotions included *fear, hostility, sadness*, and *hate* (α_Time 3_ = 0.76).

#### Esteem for Humanity

The humanity-esteem scale (Luke and Maio, 2009; Spanish version by Sevillano and Fiske, 2017) comprises ten items to measure attitudes toward humanity (e.g., “*All in all, I am inclined to regard the human species as a failure*” and “*I feel that human beings do not have much to be proud of*”; α = 0.84) using a Likert-type scale from 1 *(strongly disagree*) to 7 (*strongly agree*).

#### Abstraction as Personal Disposition

The behavior identification form (BIF, Vallacher and Wegner, 1989) is a widely-accepted scale (Burgoon et al., 2013) for measuring personal disposition to represent actions in abstract or concrete terms. Each question asks participants to describe an action (e.g., “locking a door”) by choosing an option that represents the behavior abstractly (e.g., “securing the house”) or concretely (e.g., “putting a key in a lock”). We used the original BIF scale composed of 25 items (α = 0.80). A construal level index was calculated by adding each participant’s responses, assigning 0 when the response was concrete and 1 when it was abstract. A higher BIF score represented a greater tendency to identify behaviors at a more abstract level (*Md* = 19; *M* = 18.43; SD = 4.01).

### Statistical Analyses

Repeated measures ANOVAs were calculated to test the differences between the different temporal measures of the dependent variables and between positive and negative emotional reactions at each time. We conducted independent samples *t*-tests to compare the means of the dependent variables between male and female participants. Pearson’s correlations between the BIF scores and measures were calculated to show their relationships.

To explore the influence of the construal level, we used the BIF scores to classify the participants into two groups based on the median: the abstract group presenting BIF scores ≥*Md*; and the concrete group presenting BIF scores <*Md.* Then, we conducted two-way mixed factorial ANOVA tests and independent samples *t*-tests to compare the means of emotional reactions, emotional climate, and esteem for humanity between concrete and abstract participants.

## Results

### Intensity of Vicarious Emotional Reactions

First, we tested whether empathic concern and personal distress toward Parisians varied over time. We conducted two repeated measures ANOVA at the three times, one for empathic concern and one for personal distress. We found significant differences between the three temporal measures for empathic concern, *F*(2, 504) = 258.38, *p* < 0.001, $\eta_{\text{p}}^{2}$ = 0.50, and personal distress, *F*(2, 504) = 241.43, *p* < 0.001, and $\eta_{\text{p}}^{2}$ = 0.49. All pairwise comparisons, corrected by Bonferroni, were significantly different for empathic concern and personal distress (for all, *p* < 0.001). The intensity of both types of vicarious emotions decreased over time. Empathic concern was higher in session 1, *M_1_* = 5.50 (SD = 1.16), than session 2, *M_2_* = 4.88 (SD = 1.35), and session 3, *M_3_* = 4.03 (SD = 1.46). This was also the case for personal distress: *M_1_* = 4.34 (SD = 1.19); *M_2_* = 3.73 (SD = 1.35); and *M_3_* = 2.84 (SD = 1.20).

A within-subject comparison considering both types of vicarious emotions showed that empathic concern was more intense than personal distress over time, *F*_Time 1_(1, 252) = 208.27, *p* < 0.001, $\eta_{\text{p}}^{2}$ = 0.45; *F*_Time 2_(1, 252) = 197.89, *p* < 0.001, $\eta_{\text{p}}^{2}$ = 0.44; *F*_Time 3_(1, 252) = 297.02, *p* < 0.001, and $\eta_{\text{p}}^{2}$ = 0.54.

Although the number of male (*N* = 57) and female (*N* = 196) participants was not equivalent, we calculated the influence of sex on emotional reactions using independent samples *t*-tests. The results showed that men and women reported similar empathic concern and personal distress in each temporal moment (*p*s > 0.05). See the means and standard deviations in Table 1.

**TABLE 1:** Descriptive statistics: means (SDs).

Measures and session	Complete sample (*N* = 253)	Female participants (*N* = 196)	Male participants (*N* = 57)
**Empathic concern**			
First session	5.50 (1.16)	5.56 (1.14)	5.29 (1.19)
Second session	4.88 (1.35)	4.93 (1.30)	4.69 (1.47)
Third session	4.03 (1.46)	4.09 (1.47)	3.82 (1.39)
**Personal distress**			
First session	4.34 (1.19)	4.30 (1.20)	4.45 (1.16)
Second session	3.73 (1.35)	3.72 (1.35)	3.72 (1.35)
Third session	2.84 (1.20)	2.83 (1.19)	2.85 (1.23)
**Emotional climate (third session)**			
Positive	2.47 (0.56)	2.49 (0.58)	2.40 (0.47)
Negative	2.77 (0.72)	2.77 (0.74)	2.77 (0.67)
**Esteem for humanity (third session)**	4.82 (0.86)	4.86 (0.85)	4.64 (0.87)
**BIF (third session)**	18.43 (4.00)	18.28 (3.83)	18.94 (4.58)

*No differences by sex were found. Women and men presented similar scores in all variables, p > 0.05.*

### Emotional Climate and Esteem for Humanity

Two months after the attacks, a within-subject ANOVA showed that the negative emotional climate was perceived to be higher (*M*_Negative_ = 2.77; SD_Negative_ = 0.72) than the positive emotional climate (*M*_Positive_ = 2.47; SD_Positive_ = 0.56), *F*(1, 252) = 19.98, *p* < 0.001, and $\eta_{\text{p}}^{2}$ = 0.07. Both dimensions presented a moderate level (on the five-point scale).

Correlations showed the relationships between the variables (see Table 2). In the last session, the positive emotional climate was positively related to empathic concern (*r* = 0.18, *p* = *0.004*) being uncorrelated with personal distress (*r* < 0.03, *p* = *0.63*). The correlations between the negative emotional climate and both types of vicarious emotions were significant: empathic concern (*r* = 0.15, *p* = *0.02*) and personal distress (*r* = 0.32, *p* < *0.001*). These findings support the links between interpersonal and collective emotions.

**TABLE 2:** Correlations between abstraction and measures (*N* = 253).

	Abstraction level (third session)
**Empathic concern**	
First session	0.18**
Second session	0.21**
Third session	0.22**
**Personal distress**	
First session	0.04
Second session	0.12
Third session	0.15*
**Emotional climate (third session)**	
Positive	0.15*
Negative	−0.02
**Esteem for humanity (third session)**	0.23**

*p < 0.05; **p < 0.01.

Esteem for humanity was moderately positive (*M* = 4.81; SD = 0.86) and somewhat lower than in other studies (*M* = 4.97; Sevillano and Fiske, 2017). Those participants holding a positive image of humanity perceived more positive (*r* = 0.33, *p* < 0.001) and less negative (*r* = −0.17, *p* = 0.007) collective emotional reactions than those holding a negative image of humanity. In addition, they showed more empathic concern (*r*_Time 3_ = 0.29, *p* < 0.001). This general attitude was not associated with personal distress (see Table 2). Thus, the measure of attitude toward humanity shows a stronger link with collective emotions than with interpersonal emotions.

Women and men reported similar emotional climate and esteem for humanity (*p*s > 0.05; see Table 1).

### Differences in Vicarious Emotions, Emotional Climate, and Esteem for Humanity by Level of Abstraction

The correlations between abstraction and all other measures were significant but low (see Table 2). As expected, abstraction was positively associated with greater empathic concern, a positive emotional climate, and esteem for humanity. Men and women presented similar construal level (*p* > 0.05, see Table 1).

The relationships of abstraction with negative reactions (personal distress and a negative emotional climate) were not significant, except for the positive correlation with personal distress in the last session. However, this emotion presented low intensity (*M* = 2.84; SD = 1.2).

To explore the influence of abstraction on emotional and attitudinal reactions after the terrorist attacks, we split the sample into two groups using the median BIF scores (*Md* = 19). The abstract group (*N*_abst_ = 130) showed BIF scores ≥19. The concrete group (*N*_conc_ = 123) showed BIF scores <19. Because we proposed a clear hypothesis only for positive reactions, we carried out the analysis separately for each affective valence.

**FIGURE 1:**
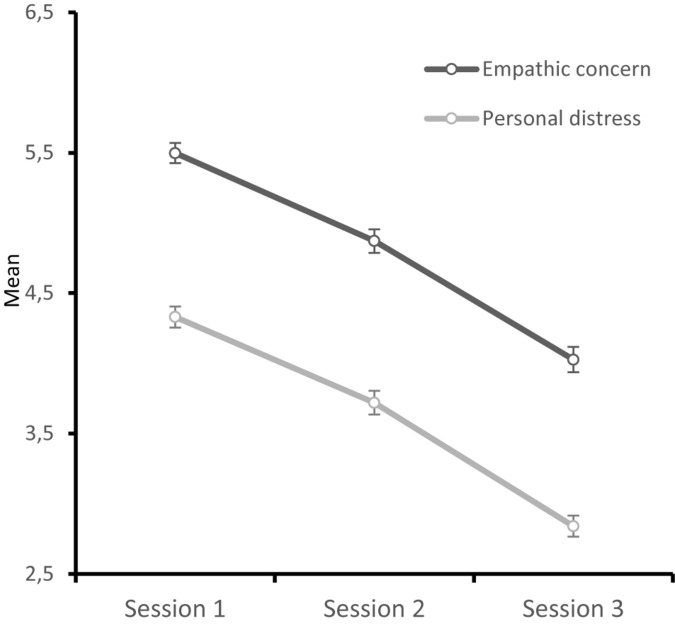
Mean ratings of personal distress and empathic concern through session.

**FIGURE 2:**
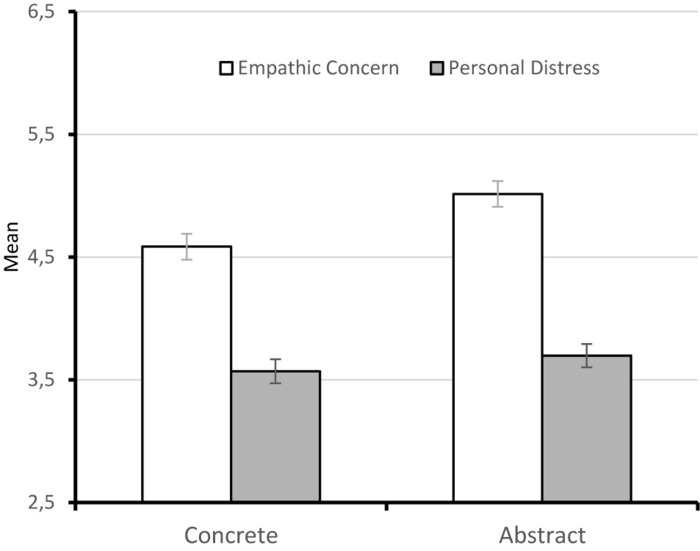
Mean ratings of empathic concern and personal distress for concrete and abstract individuals.

A two-way mixed factorial ANOVA on empathic concern with the session as the within-subject factor and the construal level (abstract individuals vs. concrete individuals) as the between-subject factor showed significant main effects for session, *F*(1.804, 452.71) = 258.93, *p* < 0.001 (Greenhouse–Geisser correction), $\eta_{\text{p}}^{2}$ = 0.51, and construal level, *F*(1, 251) = 8.55, *p* = 0.004, $\eta_{\text{p}}^{2}$ = 0.03. The interaction was not significant, *F* < 1, *p* = 0.39. Regarding the session factor, as already shown, empathic concern decreased over time: *M*_Time 1_ = 5.50 (SD = 1.16), *M*_Time 2_ = 4.87 (SD = 1.35), *M*_Time 3_ = 4.03 (SD = 1.46) [polynomial linear contrast, *F*(1, 251) = 398.00, *p* < 0.001, $\eta_{\text{p}}^{2}$ = 0.61, see Figure 1]. Regarding the construal level factor, empathic concern was higher in abstract (*M* = 5.01; SD = 1.19) than in concrete individuals (*M* = 4.58; SD = 1.16) (see Figure 2). Independent samples *t*-tests showed significant differences in empathic concern in all sessions between abstract and concrete participants, *t*s ≥ 2.29 (see Table 3).

**TABLE 3:** Descriptive statistics and construal level: means (SDs).

Measures and session	Abstract group (*N* = 130)	Concrete group (*N* = 123)
**Empathic concern**		
First session	5.66_a_ (1.05)	5.33_b_ (1.24)
Second session	5.09_a_ (1.30)	4.64_b_ (1.35)
Third session	4.27_a_ (1.46)	3.77_b_ (1.42)
**Personal distress**		
First session	4.33_a_ (1.13)	4.34_a_ (1.26)
Second session	3.80_a_ (1.28)	3.64_a_ (1.42)
Third session	2.95_a_ (1.19)	2.71_a_ (1.20)
**Emotional climate (third session)**		
Positive	2.53_a_ (0.60)	2.40_a_ (0.50)+
Negative	2.75_a_ (0.74)	2.79_a_ (0.70)
**Esteem for humanity (third session)**	4.97_a_ (0.81)	4.65_b_ (0.88)

*Means in the same row that do not share the same subscripts differ at p < 0.05. +p = 0.06.*

The same analysis was conducted for personal distress. We found that only the session was significant, *F*(1.94, 489.23) = 242.95 *p* < 0.001 (Greenhouse–Geisser correction), $\eta_{\text{p}}^{2}$ = 0.49. Personal distress decreased over time: *M*_Time 1_ = 4.34 (SD = 1.19), *M*_Time 2_ = 3.72 (SD = 1.35), *M*_Time 3_ = 2.84 (SD = 1.20) [polynomial linear contrast, *F*(1, 251) = 440.52, *p* < 0.001, $\eta_{\text{p}}^{2}$ = 0.64, see Figure 1]. No other effects were significant. Differences in the personal distress between abstract and concrete individuals were not significant, *t*s ≤ 1.56 (see Table 3).

We conducted independent samples *t*-tests to explore the influence of the construal level (abstract individuals vs. concrete individuals) on the emotional (positive and negative) climate and esteem for humanity, all of which were measured in the last session after reporting vicarious emotions toward Parisians.

The positive emotional climate was perceived to be higher, although not significantly, among abstract participants, *t*(251) = 1.87, *p* = 0.06 (see Table 3). The negative emotional climate was perceived similarly by participants with both construal levels, *t*(251) = 0.46, *p* = 0.64 (see Table 3).

Esteem for humanity was greater in the abstract group than in the concrete group, *t*(251) = 2.95, *p* = 0.003 (see Table 3).

## Discussion

Previous research has shown the confluence of negative and positive reactions to terrorist attacks (see for a review Vázquez et al., 2008). Extensive research based on the broaden-and-build theory of positive emotions (Fredrickson et al., 2003) supports the idea that emotions such as hope, love, or optimism facilitate people’s successful confrontation of adversity. Research on emotions related to resilience and coping strategies has been focused mainly on intrapersonal emotions, that is, self-focused emotional reactions felt when people experience negative events (e.g., fear and hope). In the present research, we extended these previous results to the interpersonal level, considering the vicarious emotions of empathic concern and personal distress, and to the collective level, measuring the emotional climate and esteem for humanity. This study examined emotions and attitudes in the frame of construal level theory to test how an abstract construal level promotes stronger positive reactions.

Spanish participants felt empathic concern and personal distress toward Parisians after the terrorist attacks, which are positive and negative emotional reactions involving warmth, tenderness and compassion, and worry and anxiety, respectively. At the collective level, the participants reported a moderately negative (i.e., fear, hostility, and sadness) and positive (i.e., goodness, peace, joy, hope, and solidarity) emotional climate. Specifically, perceptions of an emotional climate of hostility were overall more intense than perceptions of positive emotional climate along the three waves of the survey; however, feelings of hope, solidarity, bonanza, and confidence persisted in the context of the traumatic event. Esteem for humanity was moderate even under the traumatic circumstances of a terrorist attack.

Spaniards were not direct victims, but they were intensely moved by these terrorist acts. Supporting previous research on intrapersonal emotions in the aftermath of terrorist attacks (e.g., Fernández et al., 2009; McArdle et al., 2012; Pelletier and Drozda-Senkowska, 2016; Luhmann and Bleidorn, 2018), our results showed that the intensity of empathic concern and personal distress declined from the following week after the attacks to 2 months later. In the three phases of the survey, both types of vicarious emotions were simultaneously reported, and empathic concern was always more intense than personal distress. The highest intensities during the first session suggest that actions motivated by these vicarious emotions, such as prosocial behaviors, should be more likely during the first weeks after terrorist attacks.

The relationship between empathic concern and negative emotional climate indicates the coexistence of mixed feelings among observers of terrorist attacks. The participants who felt higher compassion and tenderness toward Parisians were as well more concerned about a threat to their society. They felt both moved by the victims and worried and fearful because of the dramatic event. The existence of mixed vicarious emotions sheds some light in the darkness of terrorist violence. This optimistic view is strengthened by the results concerning esteem for humanity. Despite the threat of terror, people reported a positive attitude toward individuals. Esteem for humanity was associated with a more positive and less negative emotional climate and greater empathic concern. These results extend the findings on the coexistence of positive and negative reactions from previous research on intrapersonal emotions to include interpersonal vicarious emotions and collective reactions.

The present study supports the beneficial role of abstractness in the aftermath of the terrorist attacks. Abstraction intensified positive interpersonal and collective reactions (i.e., empathic concern and esteem for humanity) among the observers of Paris attacks in 2015. A higher level of abstraction increased (albeit not significantly) participants’ perceptions of the positive emotional climate. Negative reactions (i.e., personal distress and negative emotional climate) were not affected by the participants’ construal level. The results indicate that abstraction has a stronger influence on positive than negative feelings. Thus, focusing on the positive side, abstract people were able to feel more intense empathic concern toward Parisians and maintain a more positive view of humanity than concrete people.

The greater resistance of abstract people’s positive emotions and attitudes to being diminished by negative situations favors better adjustment and overcoming of negative events. People with an abstract mindset tend to value reality as a whole, examining global and long-term aspects without surrendering to the current negative circumstances. Abstraction enhances people’s resilience, even under circumstances as terrible and traumatic as a terrorist attack.

### Study Limitations

As limitations, we note that our participants were psychology students studying at the National Open University in Spain. This university is focused on adult students (*M*_age_ = 35.98, SD = 9.30) who are usually working and studying simultaneously, a characteristic that makes the results slightly more generalizable to the general population, although their interest in psychology could bias the results. These limitations show the need to replicate the study in the general population. A terrorist attack is a very dramatic event that implies a global threat, indiscriminate damage, and helplessness; other threatening events should be studied to assess the strength of our results (e.g., pandemic). Although we did not find sex differences, the number of women and men was not equivalent in our sample; because of this limitation, this result should be considered with caution. Sex differences is an important variable to test in future studies on emotional reactions. A further limitation of our study is the possible demand effect introduced by questions about Parisians; a baseline should be included in future studies. Finally, given the unpredictability and immediacy of the event and the need to address the effect with the utmost promptness, we did not pre-register the hypotheses before collecting the data.

## Conclusion

These results have shown the importance of considering the influence of the construal level on vicarious and collective feelings and attitudes when exploring the aftermath of terrorist violence. Our data revealed that the higher the level of abstraction is, the greater empathic concern and esteem for humanity. All these positive reactions are associated with resilience, prosocial behaviors and promotion of social bonds. Regardless of their style of thinking, positive and negative reactions coexisted in all participants, although abstract people seem better equipped to overcome threats and difficulties, even traumatic situations such as a terrorist attack. The present may be threatening, but looking at the future with an abstract style of thinking allows people to face adversities with greater chances of personal and collective success.

To conclude, we note that although the construal level is approached as a personal tendency in this study, it is possible to modify how people represent an event. When circumstances suggest the necessity to induce an abstract style of thinking, different procedures can be used to do so. In natural settings, for example, simple messages framed abstractly (vs. concretely) have been used to successfully change the construal level (White et al., 2011). The opportunity to modify the construal level opens the possibility of promoting more positive reactions when people face terrible situations as a terrorist attack. Future research should explore programs to promote the bright side of abstraction when personal and social circumstances become difficult and negative.

## Data Availability Statement

The raw data supporting the conclusions of this article will be made available by the authors, without undue reservation.

## Ethics Statement

The studies involving human participants were reviewed and approved by Universidad Autónoma de Madrid Ethics Committee. The patients/participants provided their written informed consent to participate in this study.

## Author Contributions

All authors listed have made a substantial, direct and intellectual contribution to the work, and approved it for publication.

## Conflict of Interest

The authors declare that the research was conducted in the absence of any commercial or financial relationships that could be construed as a potential conflict of interest.
